# Eye health: what research is needed, and where?

**Published:** 2023-01-30

**Authors:** Jacqueline Ramke, Elena Schmidt

**Affiliations:** Associate Professor Of Global Eye Health: International Centre for Eye Health, London School of Hygiene & Tropical Medicine, London, UK.; Director of Evidence, Research and Innovations: Sightsavers United Kingdom, Haywards Heath, UK.

## Abstract

Research in eye health is needed to fill evidence gaps, especially in low-and middle-income countries.

Research is key to our efforts to improve eye health and has been highlighted in recent global policies. Two of the five recommendations outlined in the World Health Organization's *World Report on Vision* focused on strengthening the quantity and quality of evidence available.^1^

The *Lancet Global Health* Commission on Global Eye Health undertook a global study to identify the ‘grand challenges’ in global eye health. In this study, 470 people from 118 countries nominated and ranked the key issues that must be addressed to improve eye health at the global and regional levels.^2^^,^^3^ After a three-round process, the top five challenges in each region were identified (see http://bit.ly/3Oy4xaR) and 16 challenges were prioritised at the global level.^3^ The top 5 grand challenges globally are summarised in Figure 1.

Unfortunately, there are substantial gaps in the evidence on how to address these challenges. Evidence gap maps – a visual tool that shows the state of evidence from systematic reviews – were recently developed for cataract, glaucoma, trachoma, diabetic retinopathy, and unaddressed refractive error.^4^ These maps show that the number of systematic reviews summarising and assessing evidence relevant to eye care is growing. However, the available evidence is still dominated by clinical research (prevention, diagnosis, and treatment) and there are significant gaps in evidence about health systems related to eye care, and on how to improve access, equity, and cost-effectiveness of eye care services. In addition, there is unequal geographic representation among the studies included in most reviews, with most of the evidence being generated in Europe, the Americas, and the Western Pacific region.^5^

More research is needed to fill these evidence gaps, particularly in low- and middle-income countries. There is a need to invest more in vision impairment surveys to ensure the availability of accurate data to monitor progress towards universal eye health. Specific attention should also be given to implementation research: how to better connect people with the interventions that we know work, particularly cataract surgery and spectacles. Equally important is research which focuses on strategies that promote equity and improve access for historically underserved groups, as well as research to improve the cost-effectiveness and sustainability of eye care services.

**Baseline estimates of service coverage**

In 2020, at the 73rd World Health Assembly, all Member States committed to monitoring progress towards effective cataract and refractive error services coverage in the decade to 2030. However, many countries are without recent national baseline estimates of service coverage. Although around half the countries in the world had carried out at least one such survey between 2000 and 2020, many were conducted a long time ago or at sub-national (rather than national) level.

**Figure 1:**
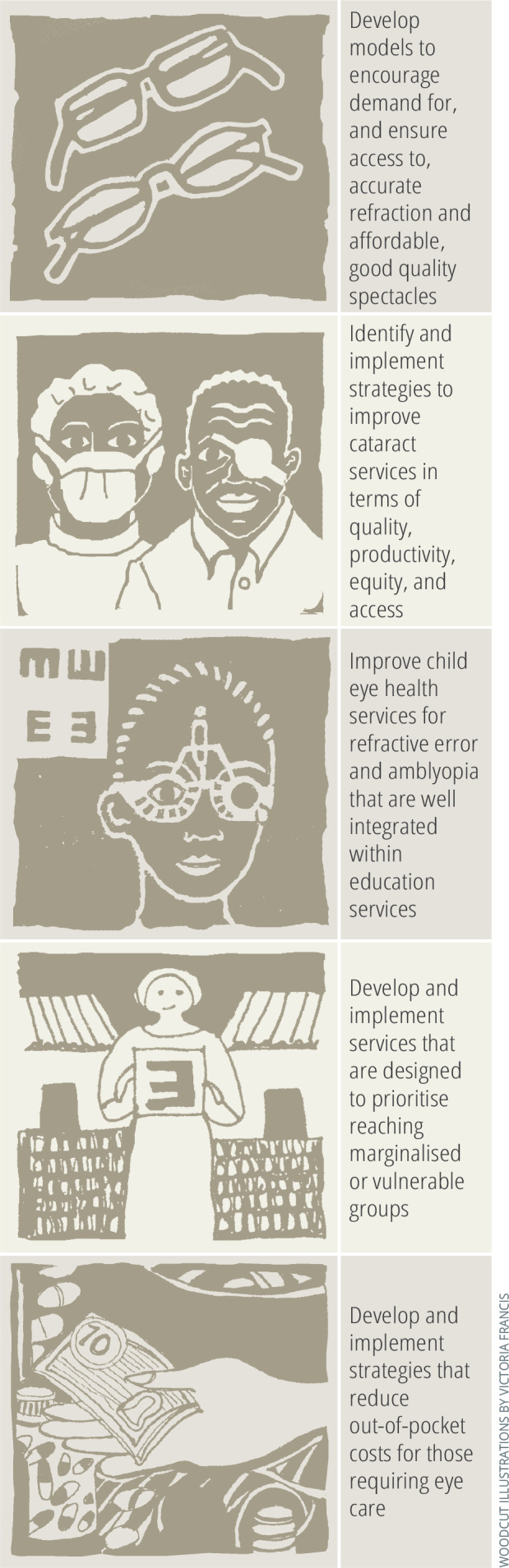
The top 5 grand challenges globally
